# Ceramide and Sphingosine-1-Phosphate in Neurodegenerative Disorders and Their Potential Involvement in Therapy

**DOI:** 10.3390/ijms23147806

**Published:** 2022-07-15

**Authors:** Cristina Tringali, Paola Giussani

**Affiliations:** Department of Medical Biotechnology and Translational Medicine, Università degli Studi di Milano, LITA Segrate, Via Fratelli Cervi, 93, 20090 Segrate, Italy; cristina.tringali@unimi.it

**Keywords:** sphingolipids, ceramide, sphingosine-1-phosphate, neurodegenerative disorders, Alzheimer’s disease, Parkinson’s disease, amyotrophic lateral sclerosis

## Abstract

Neurodegenerative disorders (ND) are progressive diseases of the nervous system, often without resolutive therapy. They are characterized by a progressive impairment and loss of specific brain regions and neuronal populations. Cellular and animal model studies have identified several molecular mechanisms that play an important role in the pathogenesis of ND. Among them are alterations of lipids, in particular sphingolipids, that play a crucial role in neurodegeneration. Overall, during ND, ceramide-dependent pro-apoptotic signalling is promoted, whereas levels of the neuroprotective spingosine-1-phosphate are reduced. Moreover, ND are characterized by alterations of the metabolism of complex sphingolipids. The finding that altered sphingolipid metabolism has a role in ND suggests that its modulation might provide a useful strategy to identify targets for possible therapies. In this review, based on the current literature, we will discuss how bioactive sphingolipids (spingosine-1-phosphate and ceramide) are involved in some ND (Alzheimer’s disease, Parkinson’s disease, amyotrophic lateral sclerosis) and their possible involvement in therapies.

## 1. Introduction

Neurodegenerative disorders (ND) are incurable diseases of the nervous system. These diseases are characterized by progressive impairment and loss of brain areas and neuronal subtypes and are associated with a gradual increase in symptoms linked to the affected brain areas. It is known that in the early stages of ND only specific subtypes of neurons degenerate, whereas in the advanced stages a more global neuronal degeneration is observed to involve different neuronal subtypes [1]. The current goals for ND treatment are early diagnosis and to slow the disease progression. Unfortunately, the understanding of the pathogenesis has not advanced to the point of effective targeted drug development.

It has been accepted that interactions between multiple environmental and genetic factors contribute to the development of ND, with aging being the most important risk factor [2]. Familiar forms of ND exist but the majority of ND are sporadic. Even if among ND many different and heterogeneous pathologies are comprised, some overlapping clinical phenotypes and molecular alterations leading to neurodegeneration have been identified. Thus, it is conceivable that common pathogenic pathways may exist. Animal model studies have contributed to our current knowledge and identified several molecular mechanisms that play an important role in the pathogenesis of ND [3,4,5,6,7,8]. In particular, the presence of extra and intra-cellular deposits of misfolded proteins and/or iron have been shown, but it is not completely clear if these aggregates are the consequence of upstream cellular defects or rather the cause of the observed dysfunctions. Alterations in systems controlling protein folding and protein degradation and removal have been observed, such as the impairment of the ubiquitin–proteosome system, autophagy, and chaperon proteins [9]. Further, mitochondrial dysfunction, leading to increased free radicals release and reduced bioenergetics, has been observed [10,11,12,13,14,15]. Oxidative stress caused by increased formation of free radicals or by impaired cellular defenses, alterations in neurotrophins release and action, neuroinflammation, malfunction of Golgi apparatus and disruption of axonal transport are other common hallmarks of ND [9]. Over many years, these dysfunctions deregulate signalling pathways such as JNK signalling, p53, Bcl-2 proteins and caspases, finally leading to neuronal death that occurs through different ways (apoptosis, autophagy or oncotic necrosis). Elucidating how neurons are driven to death may help to prevent it.

In this context and in the above cited mechanisms, lipids, in particular sphingolipids, have been demonstrated to play a crucial role [3]. Sphingolipids can originate from “de novo synthesis” to form ceramide (Cer), which is the core molecule of sphingolipid metabolism. Cer is essentially a fatty acid linked to sphingosine (Sph). Sphingomyelin is generated when the 1-hydroxyl group of Cer is linked to phosphocoline, and when a sugar or a carbohydrate chain is linked to the same position, glycosphingolipids are generated. On the other hand, Cer can be de-acylated to sphingosine (Sph) that, in turn, can be phosphorylated to sphingosine-1-phosphate (S1P) [16]. Recently, it has been demonstrated the crucial role of Cer, its metabolites and S1P in the development and progression of ND [17]. Moreover, it has highlighted the role of the S1P/Cer rheostat, in which apoptosis is promoted by increased Cer levels and concurrent with the reduction of S1P.

In this review, we will focus on bioactive sphingolipids (S1P and Cer/ceramide 1-phosphate (Cer1P)), highlighting (1) the crucial role that they have in some ND (Alzheimer’s disease (AD), Parkinson’s disease (PD), amyotrophic lateral sclerosis (ALS)), (2) the implications of their alterations in ND, and (3) their potential as targets in therapies.

### 1.1. Alzheimer’s Disease AD

AD is an age-related, non-reversible brain disorder characterized by the progressive deterioration of memory and all cognitive functions. There are three major hallmarks in the brain that are associated with the pathogenesis of AD: (1) extracellular deposits of amyloid-β (Aβ) peptides, (2) senile plaques, and (3) intracellular neurofibrillary tangles of hyperphosphorylated protein tau in the brain [18,19]. There are currently four drugs approved for the treatment of AD and the N-methyl-D-aspartate receptor antagonist. These drugs work by regulating neurotransmitters but they do not work for all patients and may help only for a limited time. Currently, there is no cure for AD, but drug treatments can delay the symptoms of the disease.

### 1.2. Parkinson’s Disease PD

One of the central pathological features of PD is the progressive loss of nigrostriatal dopamine (DA) neurons, which is accompanied by the presence of α-synuclein containing cytoplasmic inclusions, known as Lewy bodies [18]. The loss of these particular neurons causes some of the debilitating motor deficits associated with the disease, such as rigidity and bradykinesia along with some of the dysexecutive cognitive deficits [18]. Current treatments consist of dopaminergic replacement therapies that employ drugs such as the dopamine precursor l-3,4-dihydroxyphenylalanine (l-DOPA) or a dopaminergic agonist, which can improve symptoms only in early stages [18]. Several studies have shown that DA neurons derived from embryonic stem cells are capable of improving neurological features in mammalian PD models [20,21,22]. Moreover, the transplantation of human fetal mesencephalic tissue with grafted DA neurons in patients with PD promotes their survival for approximately 10 years [18].

### 1.3. Amyotrophic Lateral Sclerosis ALS

Amyotrophic lateral sclerosis (ALS) is an incurable neurodegenerative disease characterized by the selective degeneration of motor neurons in the spinal cord, motor cortex, and brainstem [23]. The etiology of this pathology is still not well known; defects in the metabolism [24], dysfunctions of T-regulatory (Tregs) lymphocytes [25], and defects in protein and RNA degradation and homeostasis [26] seem to act as major contributors to the progression of the disease. Most of the patients affected by ALS are characterized by an unknown etiology. A minority is characterized by autosomal dominant mutations in specific genes, particularly SOD1, encoding superoxide dismutase [27,28,29,30,31,32,33,34,35,36,37,38,39]. Furthermore, ALS onset appears to be associated with lipid metabolism disorders, mainly hyperlipidemia [37,38] and lipids were demonstrated to act as secondary messengers in inflammatory processes occurring during ALS development [40].

### 1.4. Sphingolipids and Nervous System

**Figure 1:**
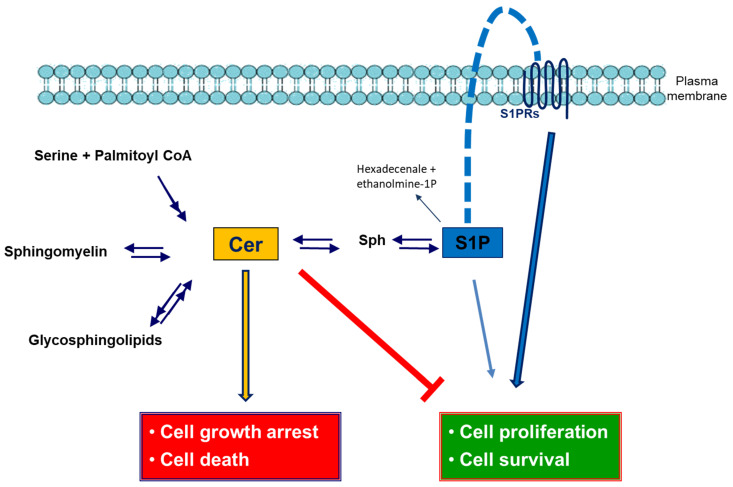
Ceramide and sphingosine-1-phospate metabolism and signaling in CNS. Ceramide (Cer) is generated from serine and palmitoyl Coenzyme A (de novo pathway) and through the acylation of sphingosine (Sph) (salvage pathway) at the endoplasmic reticulum. Cer is a substrate for sphingomyelin (SM) and glycosphingolipids synthesis at the Golgi apparatus. Sphingosine 1-phosphate (S1P) is synthesized via phosphorylation of sphingosine (Sph). S1P can be dephosphorylated back to Sph and then reacylated to Cer, or it can be degraded to hexadecenal and ethanolamine-1-phosphate (ethanolamine-1P). Many of the biological effects of S1P are mediated via specific receptors, designated S1PR.

Sphingolipids can originate from “de novo synthesis”, the degradation of complex sphingolipids, or the recycling of long chain bases through a salvage pathway (Figure 1) [41]. Their levels are tightly regulated through the enzymes responsible of their metabolism and the subcellular localization that characterize sphingolipid metabolism [41,42].

Among sphingolipids, Cer, sphingosine, S1P, and Cer1P are bioactive lipids that act as second messengers [41,43,44,45,46]. Through this function, they are involved in many cellular processes including the regulation of stress resistance, proliferation, differentiation of nervous system cells to mature phenotypes [17,47,48], cell death, senescence, adhesion, migration, inflammation, angiogenesis, and intracellular trafficking in the central nervous system [49,50]. Cer has been recognized in neurons and astrocytes as a negative modulator of cellular proliferation and survival and it is also known as an important regulator involved in the regulation of neuronal differentiation [51,52,53]. On the contrary, S1P exerts an opposite effect being a positive regulator of cellular proliferation, survival, and motility in both neurons and glial cells [54]. Moreover, in cerebellar astrocytes, S1P was demonstrated to be able to mediate calcium signaling, whereas in differentiated neurons it failed to evoke calcium signaling. This effect could be relevant to communication between neurons and glial cells in the cerebellum [55]. Increasing evidence highlights a role of sphingolipids in brain aging and ND such as AD, PD and ALS [17]. Cer1P and human Cer1P transfer protein (CPTP), that selectively transports Cer1P from the Golgi apparatus to specific cellular sites through a non-vesicular mechanism, are associated with autophagy and inflammation-related diseases such as ND [56].

More recently, animal studies and studies with human tissue samples have revealed the importance of Cer, its metabolites and S1P in the development and progression of ND. The roles of sphingolipids in neurons and glial cells are complex, cell dependent, and many times contradictory [57]. During neurodegeneration, pro-apoptotic signaling is promoted by Cer, whereas the levels of the neuroprotective S1P are reduced, indicative of the so-called S1P/Cer rheostat. The crucial role of sphingolipids during aging is also highlighted by studies performed on lower organism models such as *Caenorhabditis elegans* and yeast that have proven links between Cer synthesis and longevity [47,58,59,60,61].

## 2. Ceramide and Sphingosine-1-Phosphate in Neurodegenerative Diseases

### 2.1. Ceramide and Sphingosine-1-phosphate in Alzheimer’s Disease

Aberrant sphingolipid profiles have been observed in human AD brains, and accumulated evidence has demonstrated that changes in membrane properties induced by defective sphingolipid metabolism impair generation and degradation of Aβ [62]. Kosicek pointed out the involvement of bioactive sphingolipids from the earliest, prodromal stages of AD [63]. In several studies using post-mortem brains of AD patients, as well as in animal studies, elevated levels of Cer have been observed [64,65,66,67]. It has been shown that Aβ up-regulates the activity of sphingomyelinases (SMases) [62,64,68] by a redox-sensitive, cytosolic calcium dependent phospholipase A2-arachidonic acid pathway [62,68]. In addition, Cuttler and collaborators reported that Aβ increases Cer through increased serine palmitoyltranferase (SPT) activity [62,69]. Different mechanisms have been proposed for the stimulation of SMases activities. Butterfield and coworkers suggested that SMases are stimulated by oxidative stress that is generated by Aβ [70], whereas Grimm et al. reported that Aβ directly activates neutral (n)SMase [71]. In addition, sphingomyelin levels vary significantly among reports in the brains of AD patients [64,67,69,72,73]. Analysis performed on human post-mortem brain material to determine the level of bioactive sphingolipids demonstrated that membranes from AD patients are characterized by increased levels of long chain Cer C18:0 and C24:0, and a positive correlation with increased levels and disease severity [69]. Moreover, in the early stage of AD, levels of Cer de novo synthesis increased, in particular Cer C22:0 and C24:0 [74].

Several studies have reported that Sph is elevated in AD brains compared to normal controls [62,64,75]. Moreover, S1P levels, as well as sphingosine kinases 1 and 2 (SphK1 and SphK2) activity, decline in a region-specific manner during the course of AD pathogenesis [76]. Loss of S1P and SphK activity was demonstrated early in AD pathogenesis, and prior to AD diagnosis [76]. In addition, He et al. reported a strong inverse correlation in tissues between Aβ levels and S1P [64]. It has been shown that the Cer/S1P rheostat is linked to the production, secretion, and aggregation of Aβ and α-synuclein [17]. Further, it has been demonstrated that sphingolipids affect PI3K/Akt signaling pathway that is, in turn, involved in the regulation of metabolism, stress response, and Bcl-2 family proteins [47,77]. Moreover, S1P/Cer ratio may be involved in the regulation of autophagy; S1P–dependent autophagy is considered a pro-survival response that participates in the clearance of damaged proteins and organelles [78]. In particular, in AD, autophagy has a fundamental role in defending from damaged cellular components [79,80]. Nevertheless, autophagy can be also a cell death mechanism; in this case the autophagolysosomal degradation of mitochondria is dependent on the interaction between Cer and the microtubule-associated protein 1A/1B-light chain 3 (LC3)-II on lysosomes [81].

Amaro and colleagues suggested that plasma membrane sphingolipid lipid rafts organization could also influence Aβ aggregation [82]. It has been demonstrated that the addition of exogenous Cer, as well as high levels of endogenous Cer, increase Aβ level. Conversely, fingolimod (FTY720), a Sph analogue and S1P receptor agonist, reduces (i) hippocampal neuron damage and, as a consequence, decreases the learning and memory deficits in a rat model when it is injected in the hippocampus with pre-aggregated Aβ [83], and (ii) neuronal Aβ generation [84]. It has been shown that Aβ production correlates with low levels of ShpK1 and high levels of S1P lyase [85].

It has been shown that sphingolipids and enzymes involved in their metabolism modulate exosome secretion. nSMase2 and sphingomyelin synthase 2 (SMS2), in particular, modulate exosomes secretion that can contribute to the spread or to the clearance of neurotoxic proteins among brain cells [86,87,88].

Moreover, adiponectin regulates various metabolic functions and reduces inflammation. Adiponectin receptors (AdipoR1 and AdipoR2) are expressed in the brain [89] and have been shown to have an intrinsic ceramidase activity enhanced by adiponectin [90]. Recently Anishchal et al. demonstrated that in aged 5XFAD mouse model of AD the pattern of AdipoRs was significantly perturbed. In fact, they have shown a significant decreased expression of both AdipoRs in neurons, as well as a decrease in endothelial cells of AdipoR1, but robust expression of AdipoR2 in activated astrocytes [91]. They suggest that astrocytes may utilize AdipoR2 signalling to fuel their activated state in the AD Brain [91]. These findings suggest that the modulation of AdipoR and, as a consequence of its intrinsic ceramidase activity, can diminish amyloidogenic Aß production.

**Figure 2:**
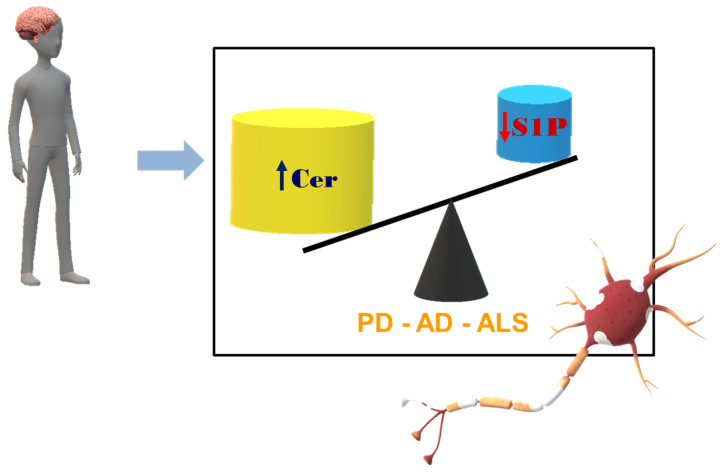
Ceramide (Cer) and sphingosine-1-phospate (S1P) levels in AD, PD, and ALS. The changes in bioactive sphingolipid levels observed in neurodegenerative disorders. The imbalance in the ratio between the concentrations of the apoptosis inducer Cer and the typically anti-apoptotic S1P.

To summarize, these reports strongly indicate that excessive levels of Aβ can alter sphingolipid profiles even if further studies are necessary to verify the contribution of Aβ-induced impairment of sphingolipid metabolism in AD. Many studies on post-mortem brain tissues highlight the imbalanced ratio between Cer and S1P, demonstrating the increase in levels of the apoptosis inducer Cer and the decrease of the typically anti-apoptotic S1P (Figure 2) [17].

### 2.2. Ceramide and Sphingosine-1-Phosphate in Parkinson’s Disease

Much research has been carried out on the relationship between altered Cer metabolism and the pathogenesis of PD. Several years ago, it was demonstrated that Cer signalling activation was able to function as a mediator of apoptosis of neurons of the substantia nigra in PD inducing oxidative stress in mitochondria [92]. It has been shown that in a specific brain region of patients affected by severe PD, there was a significant increase in the expression of enzymes involved in Cer synthesis [93]. Moreover, Cer levels have been associated not only with the pathogenesis of PD but they have also been correlated with cognitive function [94].

Mielke et al. demonstrated that Cer and monohexosylceramide levels are higher in PD patients with cognitive impairment versus controls without cognitive impairment, suggesting that their metabolism is altered in PD and their higher levels are associated with worse cognitive function [95]. Abbott et al. measured Cer level in the gray matter from post-mortem PD brains and the greatest reductions were detected in Cer (d18:1/22:0), Cer (d18:1/23:0), and Cer (d18:1/24:1) in PD relative to control [93]. In the same samples, they reported a significant decrease in levels of sphingomyelin C23:0, C24:1 and C26:1, accompanied by significant increases in C18:1 and C20:0 levels [93]. All together these results suggest that Cer metabolism could be dysregulated by events involved in PD development. On these bases, the inhibition of Cer signaling could be an important target for new therapies for PD. It has been shown that, in an in vitro PD model, SphKs and S1P lyase gene expression was significantly altered and that the cytosolic SphK1 and nuclear SphK2 activities were significantly reduced [94,96,97,98]. Several papers have shown that FTY720 prevented the loss of DA neurons in 6-hydroxydopamine-, rotenone- and acute 1-methyl-4-phenyl-1,2,3,6-tetrahydropyridine (MPTP)-induced PD cellular and animal models [99,100,101,102]. Pyszko and collaborators demonstrated that in in vitro experimental PD models, the inhibition of SphKs increases α-synuclein secretion, suppresses PI3K/Akt activation and activates the gene expression of pro-apoptotic proteins, strongly suggesting that SphK1 inhibition plays an important role in caspase-dependent apoptotic neuronal death [96]. Thus, SphKs/S1P/S1P receptor signalling has a crucial role in the pathogenesis of PD and could be a promising target to develop new therapies for PD [94]. It has been reported that in post-mortem brain tissues in PD patients Cer levels increase [17], but there are no significant data on S1P levels even if, in experimental disease models, reduced SphK activity has been demonstrated [96]. Taken together, these findings demonstrate that PD is characterized by an increase of Cer levels and a decrease of S1P levels (Figure 2).

### 2.3. Ceramide and Sphingosine-1-Phosphate in Amyotrophic Lateral Sclerosis

Recently, it has been shown that changes in sphingolipids are hallmarks of ALS [36,37,103]. Cer and glucosyl and lactosyl derivatives are significantly increased in the spinal cords of patients affected by ALS [104]. Henriques et al. demonstrated that, in the spinal cord of symptomatic SOD1G86R mice, the severity of the disease correlated with the altered levels of Cer and Sph, which in turn correlated with the expression of SphK1, S1P phosphatase 2, and with Cers (d18:1/26:0) levels [17,105]. Dodge et al. [104] found, in gray and white matter samples of the cervical spinal cord of patients with idiopathic ALS, a significant increase in the total content of Cer, and in particular the authors found that C18:0, C24:1, and C24:0 molecular species were increased. Moreover, FTY720 improved neurological scores and survival in mutant mice [17,106]. Furthermore, Dodge et al. demonstrated that ALS is characterized by a significant increase of Cer levels that are not associated with decreased activity of lysosomal enzymes catalysing its degradation [103]. Brodowicz et al. demonstrated that, in motor neurons of patients affected by ALS, Cer is mainly formed through catabolic pathways and not through the de novo synthesis. Instead, apoptosis of motor neurons in transgenic mice that reproduce ALS is associated with an increase in the activity of SMase [107]. The alteration of Cer and sphingolipids in lumbar spinal cord of transgenic mice depend on the stage of ALS [23,103]. Moreover, Weiss et al. demonstrated that Cer levels are increased in the skeletal muscles of mice. In particular, Cer (d18:1/16:0) levels are elevated in the skeletal muscles of the knock-in homozygote VCP^R155H/R155H^ mutant mice, compared to wild-type controls [108]. A lipid-enriched diet improves and reverses these defects, suggesting that dysfunctions in lipid-derived signaling could be critical to disease pathogenesis [108].

Gutner et al. demonstrated that, in transgenic mice simulating ALS, the metabolism of Sph, sphinganine and S1P is dysregulated [109]. In particular, Sph and sphinganine levels were significantly increased mainly in the spinal cord of mice but their levels did not change in brain structures during ALS development.

Differences were found in the gene expression of enzymes involved in sphingolipid metabolism [23]. The early stages of ALS are characterized by a rapid increase of S1P lyase expression that leads to apoptosis during the final stages of the disease [23]. Taken together, these findings demonstrate that ALS is characterized by an increase of Cer levels and a decrease of S1P levels (Figure 2).

## 3. Deoxysphingolipids and N-acetyl Sphingosine in ND

Recently, an important role of deoxysphingolipids in neural diseases has been highlighted [110]. The accumulation of deoxysphingolipids has been demonstrated in neural diseases such as peripheral neuropathy Hereditary and Sensory Neuropathy 1. In particular, Schwartz et al. found mutations in serine palmitoyltransferase, leading to incorporation of alanine and glycine instead of serine, and giving rise to the production deoxysphingolipids deoxydihydroceramides, deoxyceramides, and deoxysphingoid base species [111,112,113,114].

Martinez et al. demonstrated that in dopaminergic neuroblastoma cells and primary dopaminergic neurons, tumor necrosis factor (TNF)-α treatment promotes generation of Cer, and induces accumulation of atypical deoxy-sphingoid bases. They suggested that Cer and atypical deoxy-sphingoid bases may represent novel drug targets for development of neuroprotective strategies that can delay or attenuate the progressive loss of nigral DA neurons in patients with PD [115]. Moreover, Dohrn et al. demonstrated that diabetic distal sensorimotor polyneuropathy, a frequent, disabling complication of diabetes mellitus, is characterized by the elevation of 1-deoxy-sphingolipids [116]. They cross-sectionally compared the levels of 1-deoxy-sphingolipids in Diabetic distal sensorimotor polyneuropathy other nerve disorders but no elevated 1-deoxy-sphingolipids plasma levels were seen in patients with any of the other diagnoses, including ALS [116].

Recently, a novel sphinolipid, the N-acetyl sphingosine (N-AS), which is an intermediate product in Sph metabolism, has been reported to have a role in the central nervous system physiology and disease [117]. It has been shown that acetyl-CoA binds to the ATP-binding domain in SphK1 and transfers the acetyl group to Sph, leading to the production of N-AS. N-AS is synthesized both by neurons and microglia under physiological conditions but in AD the expression and the function of N-AS are affected by Aβ deposition. Ayub et al. demonstrated that in AD, N-AS levels were significantly reduced in microglia compared to neurons. During AD progression, Aβ plaques are increased, and SphK1 activity is impaired leading to decreased levels of N-AS. In microglia N-AS levels are reduced due to the reduced availability of acetyl-CoA in these cells. It has also been demonstrated that the decrease in N-AS synthesis by acetyl-CoA deficient microglia is associated to a lower acetylation of COX2 which affect the release of specialized pro-resolving lipid mediators causing poor resolution of neuroinflammation and AD pathology [117].

## 4. From Ceramide toward Complex Sphingolipids

Cer is a pivotal molecule of interchange among different pathways. It can be phosphorylated by Cer kinase to Cer1P, converted back to sphingosine by the action of ceramidase, conjugated with phosphorylcoline to form sphingomyelin, or it can be glucosylated to form glucosylceramide or galactosylated to form galactosylceramide, the precursors of complex sphingolipids.

Heterozygous defective mutations mapped on the GBA1 gene encoding the lysosomal enzyme glucocerebrosidase that is involved in the catabolism of glucosylceramide are associated with PD and dementia with Lewy bodies [118]. On the other hand, PD patients who do not carry any mutations in GBA1 gene also have a low activity of glucocerebrosidase in their substanzia nigra [119]. To explain this, dysfunctions in E3 ligase thyroid hormone receptor interacting protein 12 (TRIP12) expression were suggested because this enzyme ubiquitinates glucocerebrosidase inducing its degradation by the proteosoma [120]. Decreased glucocerebrosidase levels favor α-synuclein deposits [121]. Glucosylceramide accumulation in neurons has been demonstrated to cause neuroinflammation and neurodegeneration that ultimately lead to neuron death [122], but, strangely, even if glucocerebrosidase activity is reduced in PD, there is little evidence of an increase of glucosylceramide [123]. However, glucosylceramide along with Cer was found to be increased in cerebrospinal fluid of ALS patients [124]. Accordingly, glucosylceramide synthase, the enzyme promoting glucosylceramide synthesis, was found to be upregulated in SOD1G86R mice and in ALS patients [125].

In addition to glucosylceramide increase, ALS patients also show increased levels of galactosylceramide, lactosylceramide, globotriaosylceramide, and gangliosides in their spinal cords [104]. Thus, it could be possible to conclude that the regulation of sphingolipid metabolism is severely compromised at different steps in ALS.

In AD, alterations affecting complex sphingolipids have also been recognized and, particularly, gangliosides GM3 and GM1 have been detected at increased levels in AD brain and have been found to induce amyloidogenesis of amyloid precursor protein (APP), Aβ aggregation and the formation of plaques. Accordingly, glucosylceramide synthase inhibition was demonstrated to ameliorate cognitive functions, reducing Aβ-induced neurodegeneration and GM3 levels in murine models [126].

Lactosylceramide can be directly synthetized from glucosylceramide, or it can be derived from the catabolism of more complex sphingolipids. It is an important inducer of neuroinflammation because it is able to activate astrocytes through phospholipase A2 and RAS/ERK1/2 pathways. Moreover, it is employed for the biosynthesis of gangliosides that are frequently altered in ND [127]. Patients with PD showed increased levels of hexosylceramide and lactosylceramide [95], whereas gangliosides GM1 and GM3 have been demonstrated to promote α-synuclein aggregation [128]. Levels of GM1 and GM3 were found to be increased also in spinal cords of ALS patients [104], confirming the possible role of these specific gangliosides in neurodegeneration triggered by different cues.

Anti-ganglioside antibodies have been frequently identified in ALS, albeit with contradictory results [129,130].

## 5. Effects of Modulators of Sphingolipid Metabolism on ND Phenotypes

In light of ever-increasing data showing the correlation between sphingolipid metabolism and ND, different molecules have been proposed and are being evaluated for their possible clinical efficacy.

### 5.1. S1P Receptors and S1P Metabolism as Potential Therapeutic Targets

S1P receptor modulators have been demonstrated to be efficacious in both animal studies and in clinical trials for ND [131,132]. The oral Sph analogue FTY720 has been approved by the Food and Drug Administration and attenuates the clinical progression of multiple sclerosis (MS) [133,134]. FTY720 is phosphorylated by ShpK2 to FTY720-phosphate (FTY720-P). FTY720 is therefore a pro-drug that, after phosphorylation by SphK2 to FTY720-P intracellularly, is exported from cells and binds to S1P receptors, with the exception of S1P_2_, resulting in S1P_1_ internalization [135,136]. FTY720 is lipophilic and crosses the blood brain barrier, exerting direct neuroprotective effects in the central nervous system in addition to peripheral immuno- suppressive activity [134]. FTY720 was demonstrated to have positive effects not only for demyelinating disorders but also for different neurological diseases such as AD [99]. Furthermore, Aβ load is decreased in APP/PS1 mice by the inhibition of β-secretase when treated with FTY720, possibly by modulating the transport of Aβ through the blood brain barrier [137]. Moreover, FTY720 has also been investigated as a treatment for other ND, such as PD, because of its anti-inflammatory and neuroprotective effects [138]. There are certain limitations of FTY720 therapy due to side effects in patients with cardiovascular and immunological risk factors [131,139,140]. For this reason, during the last few years, second generation compounds, such as Siponimod, KRP-203, CS-0777, and RPC-1063, with structures similar to FTY720, have been synthesized. These compounds have been used in clinical trials [99,141] but, to date, FTY720 is the only S1PR modulator used clinically.

As described above, changes in S1P levels occur during AD pathogenesis. (S)-Methyl 2-(hexanamide)-3-(4-hydroxyphenyl) propanoate (MHP), a novel synthetic SphK1 activator that increases S1P levels, significantly suppressed invasion of the virulent *Staphylococcus aureus* into murine skin explants [142]. MHP is a possible therapeutic able to limit the progression of AD stimulating innate immunity.

### 5.2. Cer metabolism as Potential Therapeutic Target

Ambroxol acts as a chaperone able to stabilize and help the folding of the enzyme glucocerebrosidase and to favor its transport to lysosomes. It has been studied as an expectorant cough drug for many years and only recently a novel function has been identified that could be relevant for the treatment of PD patients. Ambroxol crosses the blood–brain barrier and was demonstrated to reduce α-synuclein levels in cerebrospinal fluids of both patients with and without GBA1 mutations [143]. In addition, ambroxol also inhibits the product of the GBA2 gene, which is the non-lysosomal glucosylceramidase, localized on the endoplasmic reticulum. GBA2 was found to be increased in the spinal cords of SODG86R mice models of ALS, whereas treatment with ambroxol delayed the appearance of the first motor impairments, prevented muscle denervation and promoted axonal plasticity [144]. In light of these results, the use of this drug for ALS patients has been proposed.

The inhibition of Cer synthesis through myriocin in SH-SY5Y cells uploaded with α-synuclein promoted the degradation of protein aggregates, triggering autophagy [145]. Similarly, myriocin improved the outcome and the survival of mice models of motor neuron degeneration [146], further demonstrating that the modulation of sphingolipid metabolism could be effective in the treatment of neurodegeneration. Moreover, other compounds that inhibit serine palmitoyl transferase such as (i) L-cycloserine, which blocks Aβ production in neurons and astrocytes [147] and (ii) ARN14494, which prevents the synthesis of pro-inflammatory cytokines TNF-α and interleukin (IL1)β, transforming growth factor TGFβ1, and oxidative stress-related enzymes iNOS and COX2 in mouse primary cortical astrocytes [148]. ARN14494 also has neuroprotective properties in primary cortical neurons. All together these data suggest that serine palmitoyl transferase inhibition could be a safe therapeutic strategy for AD.

nSMase2 may also be a viable therapeutic target to inhibit AD progression. Phenyl (R)-(1-(3-(3,4-dimethoxyphenyl)-2,6-dimethylimidazo[1,2-b]pyridazin-8-yl)-pyrrolidin-3-yl)carbamate 1 (PDDC), an nSMase2 inhibitor, was reported to have significant improvement cognition in 5XFAD mice, a mouse model of AD. Moreover, cambinol, a potent nSMase2 inhibitor, significantly decreased cell death in primary neurons promoted by TNF-α or IL1β through Cer increase [149].

## 6. Perspectives and Concluding Remarks

Sphingolipids are not only abundant structural components of the plasma membrane of nerve cells, but are also deeply involved in nervous system development and in the development and progression of ND [17,46,47,48,49,150]. Sphingolipids, S1P, and Cer play a crucial role in ND, in particular in AD, PD, and ALS. During neurodegeneration, Cer-dependent pro-apoptotic signaling is promoted, whereas the levels of the neuroprotective S1P are reduced. Moreover, changes affecting complex sphingolipid levels have been recognized in AD, PD, and ALS. The role that S1P and Cer have in the regulation of signaling pathways involved in the regulation of cell fate modulating processes, such as cell proliferation or death and cell differentiation [46], highlights their importance in the development of the nervous system, and the changes in their metabolism supports the involvement of these molecules in the development and progression of ND.

The findings that altered sphingolipid metabolism has a role in neurodegenerative diseases suggest that the modulation of sphingolipid metabolism might provide a useful strategy for treating these disorders [46,94]. Furthermore, ND are associated with mutations able to impair membrane trafficking between endosomes and the Golgi apparatus. Petit et al. demonstrated that altered sphingolipid metabolism causing Cer level increase, due to altered traffic between endosomes and Golgi apparatus, contributes to neurodegeneration but the inhibition of Cer synthesis by myriocin could be an important strategy for treating ND [146].

S1P has a crucial role in the regulation of physiological and pathological conditions. On these bases, SphKs or/and S1P receptor inhibitors might be very promising tools for the treatment of neurodegenerative diseases [151,152]. The very promising preclinical data of FTY720 sets up the framework to use more selective S1P receptor inhibitors [153]. Furthermore, another strategy to modulate S1P signaling could be the inhibition of the S1P transporter Spinster2 (SpnS2) even though, to date, no inhibitors are available for Spns2.

It was recently highlighted that sphingolipids and the enzymes for their metabolism are involved in the biogenesis and release of extracellular vesicles that might participate in the spread of neurotoxic molecules between brain cells, or in their clearance [17,88]. Lombardi et al. demonstrated that vesicular S1P contained in the extracellular vesicles stimulates oligodendrocyte precursor cells migration, a fundamental step in myelin repair [154]. The secretion of extracellular vesicles might be exploited to develop novel therapeutic approaches.

## Data Availability

Not applicable.
